# Novel Nanocarriers for Targeted Topical Skin Delivery of the Antioxidant Resveratrol

**DOI:** 10.3390/pharmaceutics12020108

**Published:** 2020-01-29

**Authors:** Christofori M. R. R. Nastiti, Thellie Ponto, Yousuf Mohammed, Michael S. Roberts, Heather A. E. Benson

**Affiliations:** 1 School of Pharmacy and Biomedical Sciences, Curtin Health Innovation Research Institute, Curtin University, Perth, WA 6845, Australia; c.nastiti@postgrad.curtin.edu.au (C.M.R.R.N.); thellie.ponto@postgrad.curtin.edu.au (T.P.); 2 Faculty of Pharmacy, Sanata Dharma University, Yogyakarta 55282, Indonesia; 3 Therapeutics Research Centre, Faculty of Medicine, The University of Queensland Diamantina Institute, Woolloongabba 4102, Australiam.roberts@uq.edu.au (M.S.R.); 4 School of Pharmacy and Medical Sciences, University of South Australia, Adelaide 5000, Australia; 5 Therapeutics Research Centre, Basil Hetzel Institute for Translational Medical Research, The Queen Elizabeth Hospital, Adelaide 5011, Australia

**Keywords:** nanocarriers, targeted delivery, skin permeation, resveratrol, antioxidant

## Abstract

Resveratrol (RSV) is a potent lipophilic antioxidant with a low aqueous solubility. Novel nanoformulations have been successfully developed and evaluated to increase the potential of resveratrol as a skin targeting antioxidant. Nanoformulations were prepared using a spontaneous emulsification method, and characterized and evaluated for their capabilities to penetrate/permeate the skin. In nanoformulations, the thermodynamic activity of the RSV penetration into/permeation through the skin was correlated with the thermodynamic activity of the RSV in the formulations. When terpenes were incorporated into the nanoformulations, the permeation of RSV through the skin increased and correlated with an increasing lipophilicity of the terpene. The nanoemulsion containing eugenol showed the highest RSV penetration into the stratum corneum (SC) and the epidermis-dermis-follicle region, whereas the limonene containing nanoemulsion had the highest RSV permeation through the skin (the enhancement ratios, compared to a saturated solution of RSV, were (i) 9.55 and (ii) 12.61, respectively, based on the average RSV amount (i) in each skin region and (ii) permeation through skin).

## 1. Introduction

Resveratrol (E-5-(4-hydroxystyryl) benzene-1,3-diol; RSV) is a potent natural polyphenolic antioxidant [1] that can be extracted abundantly from grape skin and seeds, berries, peanuts, and red wine. RSV has gained much interest due to its potential to generate a range of therapeutic effects and the suggestion that it is a contributing factor in the so-called “French paradox”: the observed reduced risk of coronary artery disease in French people attributed at least in part to the regular consumption of red wine [2]. There is good evidence for RSV supporting the heart function, protecting against cardiovascular diseases [3], neuro-disorders [4,5], diabetes [6], and cancer [7], due to its antioxidant and anti-inflammatory effects. Of particular relevance to this article, RSV has demonstrated a potential for antiaging effects and for protecting against UV damage in the skin due to its antioxidant and collagen stimulating activity [8,9].

Whilst RSV is relatively well absorbed orally (approximately 70%), it is subject to an extensive first pass hepatic elimination resulting in poor oral bioavailability [10]. The direct application to the skin is an attractive administration route, as the RSV metabolism in skin is significantly lower than that in the liver [11]. A topical application is particularly appropriate where RSV is being administered for the antiaging of skin or other dermatological or cosmeceutical purposes. 

**Figure 1:**
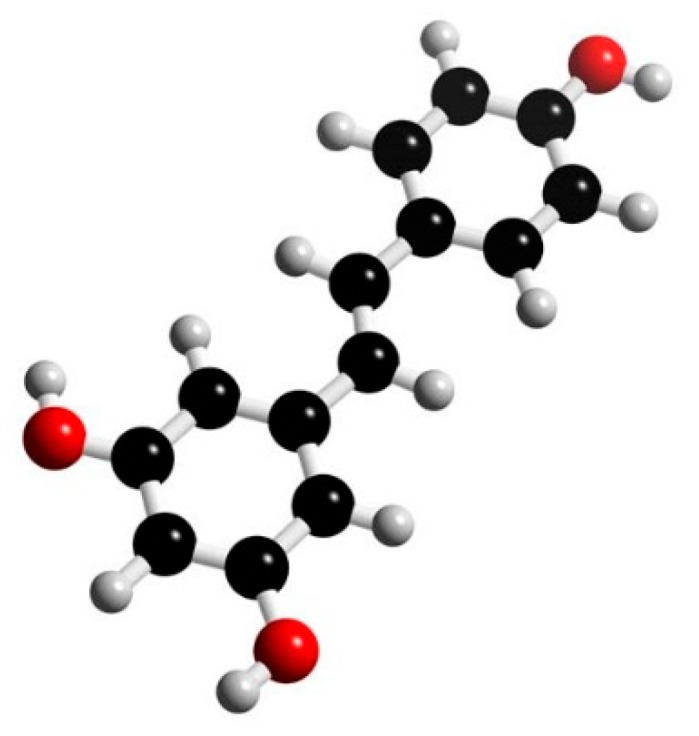
The structure of resveratrol (RSV). Adapted from © Karl Harrison 3DChem.com.

RSV (MW: 228.25; Figure 1) has a poor aqueous solubility (50–60 µg/mL) [12], and is unstable in the presence of UV light and basic conditions, thus providing a formulation challenge. The skin permeation is low; for example, Hung et al. [13] reported that the RSV flux from a saturated solution in PBS pH 6 through female nude mouse skin was 1.59 ± 0.08 nmol/cm^2^/h. To achieve the targeted topical delivery, there is a need to increase the solubility of RSV in the formulation and stratum corneum (SC), enhance the RSV stability, and increase the diffusion of RSV through the SC and deeper epidermal layers.

A range of approaches have been investigated to improve the topical delivery of RSV, including micro/nanoemulsions [14], niosomes [15], ethosomes [16], nanosponges [17], microparticles [18], and nanoparticles [19]. Nano/microemulsions provide particular advantages for a topical product formulation because they are transparent optically isotropic and kinetically stable colloids [20]. 

Micro- and nanoemulsion formulations have properties that suggest they have potential for the successful skin delivery of RSV [20,21]. Micro- and nanoemulsions consist of an oil phase, surfactant, cosurfactant, and aqueous phase, which create an isotropic, transparent/translucent, single-phase system of nano-sized droplets [20]. They have an excellent solubilizing capacity for lipophilic compounds, provide protection for relatively unstable molecules, simplicity in fabrication, and a good stability [20]. Juškaitė et al. [22] developed an RSV microemulsion containing ethyl oleate (oil phase), PEG-8-caprylic/capric glycerides (surfactant), polyglyceryl-6-isostearate (co-surfactant), and water. The highest penetration in human skin was achieved using a formula with a S_mix_ ratio of 5:1 (1.96 ± 0.41µg/cm^2^). However, the concentration of both surfactant/cosurfactant in the formula was higher than 45%, which might increase the skin irritation potential [23]. The pH of the optimized formulations (7.01–7.15) is above the ideal skin pH range, and as RSV is more stable in an acidic environment [12] this is also a limitation of the developed formulation. 

In the current study, we develop self-assembly and stable nanoemulsions for RSV skin delivery that can solubilize RSV with a relatively low composition of oil, surfactant-cosurfactant, and that can provide a good skin penetration and permeation of RSV. The nanoemulsions also protect RSV for a long duration of storage.

## 2. Materials and Methods 

### 2.1. Materials

Resveratrol (CAS# 501-36-0), was purchased from PCCA (99% purity, PCCA, USA). Kolliphor^®^ RH 40 (Polyoxyl 40 castor oil - CAS# 61788-85-0), Triacetin (CAS# 102-76-1), eugenol (CAS# 97-53-0), D- limonene (CAS# 5989-27-5), and eucalyptol (CAS# 470-82-6) were purchased from Sigma-Aldrich (North Ryde, Australia). Labrasol^®^ (PEG-8 Caprylic/Capric Glycerides - CAS# 85536-07-8) and Transcutol^®^ (Ethoxydiglycol - CAS# 111-90-0) were gifts from Gattefossé (Saint-Priest, France). Orthophosphoric acid, hematoxylin, eosin and ethanol were purchased from Thermo Fisher Scientific (Scoresby, Australia). Sodium hydroxide and sodium chloride were purchased from Chem-Supply Pty Ltd. (Gillman, Australia) and acetonitrile HPLC grade from Thermo Fisher Scientific (Scoresby, Australia). Deionised water was passed through a Milli Q apparatus (Millipore Corporation, Bedford, MA, USA).

### 2.2. Nanocarrier Development Strategy

Nanocarriers were developed based on the strategy of enhancing the solubility and incorporating materials to enhance the skin permeation, including the terpene permeation enhancers eugenol, D-limonene, and eucalyptol. We defined the quality target profile attributes (QTPP) for the liquid nanoemulsions as a clear appearance, improved stability, and optimal skin penetration-permeation. To fulfil the QTPP criteria, the critical quality attributes (CQAs) were determined both as physical characteristics and the profile of skin penetration and permeation, including the globule size, solubility, viscosity, stability, amount of RSV retained in skin, and RSV flux through skin. 

### 2.3. Formulation of Nanoemulsions

A spontaneous emulsification method was used to generate the nanoformulations with the aid of mild agitation at room temperature based on Pund et al. [24] with some modifications. The mnemonic system was applied to name the formulations, and each has also been defined in Table 1. Triacetin was selected as the oil phase, Kolliphor^®^ RH 40 and Labrasol^®^ as the surfactants, and Transcutol^®^ as the cosurfactant. PBS pH 6 was applied as the aqueous system, as RSV is more stable in an acidic environment with pH 5–6 [12], which is close to skin pH [25], thus reducing the irritation potential. 

The TKLT2P nanoemulsion system included triacetin, Kolliphor^®^ RH 40, Labrasol^®^, Transcutol^®^, and PBS pH 6. It was made by mixing the oil phase with the mixture of surfactant and cosurfactant prior to the aqueous phase addition. TKLT2P applied a surfactant-cosurfactant ratio (S_mix_) of 2:1 and ratio of oil to S*_mix_* of 1:2, with PBS pH 6 as the aqueous phase; thus, the formulation was represented by the mnemonic TKLT2P (Triacetin, Kolliphor^®^ RH 40, Labrasol^®^, Transcutol^®^ as the oil phase in a ratio of 1:2 with PBS pH 6 as the aqueous phase). The aqueous phase addition was carried out until the system started to show translucency. All processes were conducted at room temperature. RSV was loaded in the formula at a concentration of 2% (*w*/*w*). The full range of nanoformulations developed and characterised are summarised in Table 1. Based on the characterization results, one nanoformulation was chosen and further developed, with the addition of three different terpenes (eugenol, d-limonene, and eucalyptol) as chemical penetration enhancers (Table 1). All RSV nanoformulations were kept out of light throughout the formulation and characterization processes. 

### 2.4. Physical Characterization and Stability Evaluation

The RSV nanoformulations were initially characterized for physical appearance (clarity), RSV solubility, pH (MColorpHast™-Merck, Darmstadt, Germany), viscosity (Bohlin Visco 88, Malvern, Worcestershire, UK), and refractive index (Atago Refractometer, Bellevue, WA, USA). The globule size and polydispersity index were examined using a Zetasizer Nano™ ZSP (Malvern, Worcestershire, UK) after a 4-time aqueous dilution of blank nanoformulations.

Cryo-scanning electron microscopy (cryo-SEM) was performed by loading NEs into copper rivets and plunge-freezing them in liquid nitrogen slush at a temperature of −180 °C. The samples were quickly transferred into the cryo-stage (Alto 2500, Gatan, Inc., Pleasanton, CA, USA) of the microscope (JSM-6700F, JEOL Ltd., Tokyo, Japan) under vacuum. After fracturing the sample with a knife, it was viewed at −140 °C and an accelerating voltage of 2 kV.

The stability of RSV nanoformulations was evaluated based on the physical appearance and RSV quantity during the storage under a range of conditions sealed in amber or clear glass vials: (i) protected from light at an ambient temperature (22–25 °C) for one month; (ii) no light protection at an ambient temperature (22–25 °C) for one month; (iii) protected from light at 2–5 °C for one month; with an analysis of RSV in the initial samples (day 0) considered as 100% potency. The long term stability of the RSV nanoformulations was also assessed after 5 to 8 months storage. 

### 2.5. In Vitro Skin Penetration/Permeation Study

An in vitro penetration/permeation study was performed using skin obtained from newborn Yorkshire pigs which died due to natural causes. The skin was removed from the body, and the subcutaneous tissue was carefully removed using a scalpel. The hairs were reduced using Veet™ cream applied for 10 min prior to removal. The skin was then rinsed thoroughly to remove dirt and cream, then blotted dry prior to storage in the −20 °C freezer. Three different piglets were used for each experiment to provide 4–6 replications.

#### 2.5.1. Experimental Set Up

Full thickness excised skin was thawed at room temperature. The thickness of the skin was measured using a digital Vernier calliper (Kincrome, Australia) before the experiment. Skin of 400–600 µm thickness was used. The skin was mounted in Franz-type diffusion cells (SC side up), and the skin integrity was tested by electrical resistance using a digital multimeter (UNI-T^®^, Opava-Předměstí, Česko) with PBS pH 7.4 at 35 °C in the donor and receptor compartments. The PBS pH 7.4 in the donor compartment was then discarded, and the receptor compartment was filled with 20% ethanol in PBS pH 6, stirred continuously with a magnetic stirrer, and maintained in a water bath at 35 °C (skin surface temperature 32 °C). One gram of RSV nanocarrier or RSV-saturated aqueous solution (SS-control) was applied to the donor compartment (infinite dose), and the full receptor volume samples were removed for HPLC analysis and total receptor replacement with pre-warmed solution. 

#### 2.5.2. Skin Distribution Study

After completing the sampling at 8 h, tape stripping was conducted to assess the amount of RSV in the SC. Adhesive stripping tapes (22 mm diameter: CuDerm D-Squame^®^, Dallas, Texas, USA) were applied ten times on the surface of each piece of skin with a pressure of 225 g/cm^2^ using a D-Squame disc applicator (modified from Davies et al. [26]). The first two tapes were kept aside for a mass balance study, and the remaining tapes were used to determine the RSV penetrated into the SC. The skin was then sectioned prior to the RSV extraction. RSV in the tapes and sectioned skin were extracted using the mobile phase with the aid of magnetic stirring at room temperature for 3 h, prior to the determination of RSV content by HPLC.

### 2.6. HPLC Assay of Resveratrol

The resveratrol in the samples was determined using a validated HPLC assay on an Agilent™ 1200 system (Agilent Technologies, Waldbronn, Germany) consisting of a degasser, binary pump, autosampler system, variable wavelength detector at 307 nm, and Chemstation Rev B.03.01. An Apollo C18 5µ column, 150 mm x 4.6 mm (Grace Discovery Sciences, Columbia, MD, USA), was used to perform the isocratic separation with the mobile phase of acetonitrile: water: phosphoric acid = 50:50:0.05 at a flow rate of 1 mL/min.

### 2.7. Data Analysis

The cumulative amount per area (Q, µg/cm^2^) versus time (t) was plotted for the in vitro skin permeation study and used to determine the steady state flux (*J_ss_*), maximum flux (*J_max_*), lag time, and enhancement ratio (ER). *J_ss_* (μg/cm^2^/h) is determined from the slope of the linear portion of the cumulative amount (Q) versus time (t) plot:

*J_SS_* = *k_p_* × *C_v_* (1)

*J_max_* is the RSV flux of a saturated solution and can be estimated from the experimental steady state flux corrected for the known solubility in the formulation:

*J_max_* = *J_ss_* x S_v_/C_v_ (2)

where S_v_ is the saturated solubility of RSV in the vehicles (formulations), and C_v_ is the donor concentration.

The lag time was calculated based on the extrapolation of the linear portion of the cumulative amount/area vs. time plot (y = 0) as:

lag time = –(intercept of the graph)/slope (3)

### 2.8. Statistical Analysis

The data are presented as the mean ± SD (physical characteristics-related measurements) and mean ± SEM (biological system-related experiments). Normally distributed data was analysed by ANOVA or unpaired t test; a Wilcoxon test and Kruskal Wallis was applied to non-parametric data. Significant differences were considered if *P* < 0.05. All data were analyzed using GraphPad Prism™ 8 software (GraphPad Software Inc., San Diego, CA, USA). Comparisons were made between the ME formulations and controls, as well as between the different NE formulations for all the permeation parameters (*Q_24_*, *J_SS_, J_max_*) and for other experimental parameters related to the physicochemical characterization of the MEs. 

## 3. Results

### 3.1. Physical Characteristics of RSV Nanoformulations

All formulations were highly fluid and homogeneous, based on visual inspection. The viscosity of the blank nanoformulations ranged from 1.627–0.093 dPas, in the following order: ETKTP > E5K30TP > TKLT2P > E5K20TP > TKTP. All nanoformulations were light brown in colour, associated with the RSV loading, with the eugenol-incorporated formulations slightly darker due to the eugenol colour. TKLT2P was the only formulation that contained Labrasol and also the only formulation that was translucent in appearance; all others were transparent. The physical characteristics are summarised in Table 2 and Table 3.

**Figure 2:**
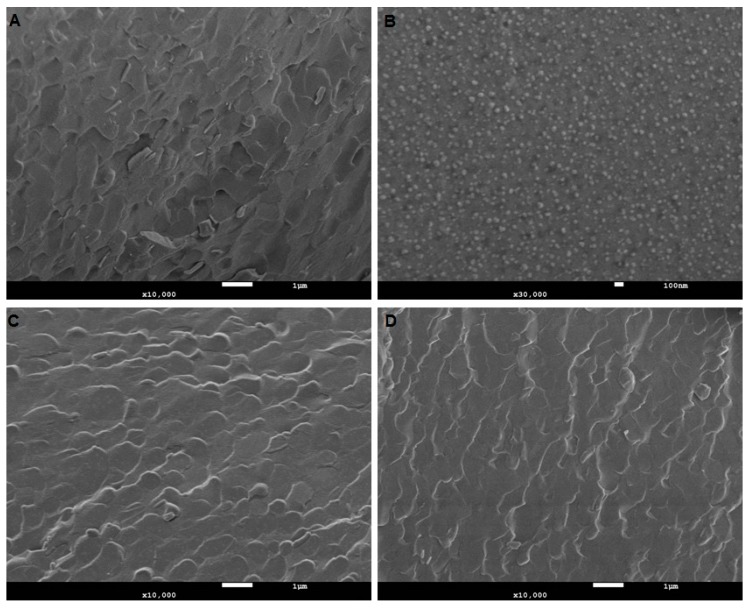
Microstructural assessment of nanoemulsion. (**A**) TKTP demonstrates a typical emulsion microstructure comprising of lipid and aqueous regions. (**B**), at a higher magnification (30,000X) to capture the globule size in ETKTP, demonstrates a lower nanometer size range as shown by DLS. (**C**) and (**D**) illustrate unloaded and loaded nanoemulsion LKTP. No changes in the microstructure after loading with the drug were visible. The white scale bar denotes 1 µm in (**A**), (**C**) and (**D**), and 100 nm in (**B**).

The CryoSEM images below (Figure 2) illustrate the microstructure of the formulated products. The aim here was to assess the innate microstructure of these semisolid formulations visually, without introducing any changes to the microstructure. Caution needs to be exercised as imaging delicately frozen semisolids of low viscosity using CryoSEM can often lead to unintended artifacts. The water component of the formulation can undergo rapid transformation during freezing, leading to structures that could easily be misinterpreted. Hence, the imaging performed here is generally a low energy regimen. This is useful in gaining complementary information, which sizing by a zetasizer does not provide.

All nanoformulations had globule sizes under 17 nm with a PDI of less than 0.250, indicating that they were monodisperse. The variation of the vehicle components and addition of terpenes did not significantly change the globule size or distribution. The size determination by cryoSEM confirmed the Zetasizer measurements (Table 3).

### 3.2. Effect of Modifying the Oil Phase and Surfactant Composition

The initial tested formulations did not contain terpenes. The TKLT2P and TKTP formulations both contained Triacetin, Kolliphor, and Transcutol, but in different proportions. Labrasol was only present in TKLT2P. As a result of the reduction in the oil phase and surfactant to 5% and 20% respectively, the TKLT2P translucency changed to give a transparent TKTP formulation. The solubility of RSV in TKLT2P was 177.16 ± 25.95 mg/mL, which was 3–4 times higher than TKTP (44.771 ± 4.159 mg/mL: Table 2).

**Figure 3:**
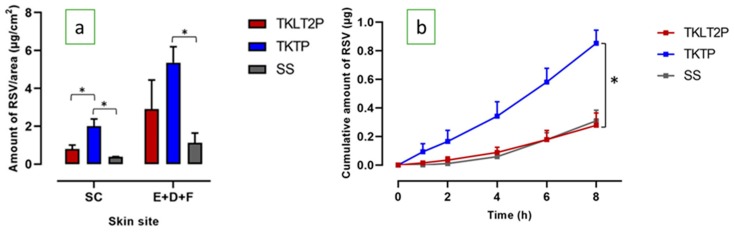
(**a**) The skin penetration and permeation profiles of RSV from the TKLT2P and TKTP formulations: the distribution of RSV in the SC and (E+D+F); (**b**) the cumulative amount of RSV after 8 h of permeation through the skin (mean ± SEM; *n* = 5-6; * *P* < 0.05).

The deposition of RSV in the SC and epidermal-dermal-follicular (E+D+F) region was significantly higher from both emulsion formulations compared to the saturated solution (SS) (Figure 3a). The amount of RSV from TKTP distributed on the SC was significantly higher than from TKLT2P (1.998 ± 0.383 and 0.805 ± 0.208 µg/cm^2^). While the amount of RSV in the (E+D+F) from TKTP was twice that of TKLT2P, the difference was not statistically significant (5.359 ± 0.845 and 2.915 ± 1.523 µg/cm^2^: Figure 3a, Table 4). 

The permeation of RSV through piglet skin was significantly increased for TKTP compared to TKLT2P (Figure 3b: cumulative amount 0.853 ± 0.091 µg and 0.278 ± 0.086 µg, respectively; *P* < 0.05). The steady state flux of RSV was three times faster from TKTP than from TKLT2P (0.103 ± 0.006 and 0.038 ± 0.010 µg/cm^2^/h), although the lag time was similar.

As TKTP appeared to be the more promising nanoformulation for RSV based on good skin penetration and permeation characteristics, this formulation was the basis for a further development, including the addition of terpene permeation enhancers. 

### 3.3. Incorporation of Eugenol: Effect on Physical Characteristics and Skin Delivery

The addition of eugenol (5%) to TKTP generated a cloudy formulation. The surfactant content was therefore increased to achieve a transparent formulation. This required an increase to 30% in the concentration of Kolliphor^®^ RH 40 surfactant to provide RSV nanoformulations with a good clarity. However, a consequence of increasing the Kolliphor concentration was that the viscosity increased to 1.621 ± 0.119 dPas, which was 16x times that of TKTP (*P* < 0.05; Table 2). This created problems for the RSV solubility determination, as the centrifuge failed to separate undissolved RSV.

**Figure 4:**
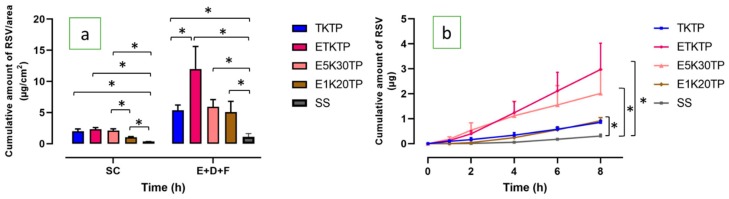
(**a**) The skin penetration and permeation profiles of RSV: the distribution of RSV in the SC and (E+D+F); (**b**) the cumulative amount of RSV after 8h of permeation through the skin (mean ± SEM; *n* = 5–6).

Figure 4a shows the comparison of RSV skin penetration of TKTP and terpene-based nanoformulations at 8 h. The amount of RSV in the SC and (E+D+F) from the nanoformulations was significantly higher than that from the RSV-saturated aqueous solution (Table 4). A similar result was also observed in the permeation of RSV through the skin after 8h (Figure 4b). The addition of eugenol and Kolliphor RH 40 (ETKTP) resulted in the highest amount of RSV retained in the skin among all nanoformulations. There was a 2.5-fold increase in the amount of RSV in the (E+D+F) from ETKTP (12.000 ± 3.598 µg/cm^2^) compared to TKTP (*P* < 0.05). ETKTP also showed a significantly higher permeation of RSV (*P* < 0.05) compared to TKTP (Figure 4b). The cumulative amount and steady state flux of ETKTP was 2.973 ±1.051 µg and 0.358 ± 0.125 µg/cm^2^/h, compared to 0.853 ± 0.091 µg and 0.103 ± 0.006 µg/cm^2^/h for TKTP. The lag time was similar for ETKTP and TKTP, with both having approximately half the lag time of the RSV-saturated aqueous solution.

To provide a mechanistic examination of these results, we then observed the interaction of eugenol and triacetin. E5K30TP contained only eugenol as the oil phase without the presence of triacetin. E5K30TP was similar in appearance to ETKTP: a light-brown, single phase, transparent appearance with a similar refractive index but lower viscosity (Table 2). The size and PDI of E5K30TP was also slightly lower than ETKTP (Table 3). 

The distribution of RSV in the SC from E5K30TP (2.104 ± 0.297 µg/cm^2^) and ETKTP (2.342 ± 0.269 µg/cm^2^) was similar (Figure 4a, Table 4), but the amount in the (E+D+F) region was significantly lower (5.914 ± 1.169 µg/cm^2^: *P* < 0.05) compared to ETKTP. There was no significant difference in the cumulative amount of RSV permeated through the skin over 8 h from ETKTP and E5K30TP (Figure 4b). 

To investigate the effect of the concentration of eugenol and surfactant on the quality of nanoformulations, the concentration of eugenol and surfactant was reduced to 1% and 20%, respectively, to generate E1K20TP. The blank nanoformulation (no RSV) appeared to be transparent with a refractive index of 1.3747 ± 0.0003 and a low viscosity (0.099 ± 0.013 dPas). The solubility of RSV in the system was 34.092 ± 1.133 mg/mL.

When applied to piglet skin, the amount of RSV in the SC from E1K20TP was 1.022 ± 0.129 µg/cm^2^, which was approximately 50% that of E5K30TP (2.104 ± 0.297 µg/cm^2^: *P* < 0.05), but the amount in the (E+D+F) was similar for both nanoformulations (Figure 4a, Table 4). 

The cumulative amount of RSV permeated over 8 h and steady state flux from E1K20TP (0.918 ± 0.126 µg; 0.142 ± 0.017 µg/cm^2^/h) had a lower trend with the decreased surfactant content in the nanoformulation, but these differences were not statistically significant. 

### 3.4. Incorporation of Limonene and Eucalyptol: Effect on Physical Characteristics and Skin Delivery 

The incorporation of 1% eugenol, D-limonene, and eucalyptol yielded transparent formulations with excellent clarity and similar physical characteristics in terms of appearance, RSV solubility, viscosity, and refractive index (Table 2), but small differences in the globule size (LKTP 15.73 ± 0.07 nm; EuKTP 14.54 ± 0.04 nm; E1K20TP 13.84 ± 0.01 nm).

**Figure 5:**
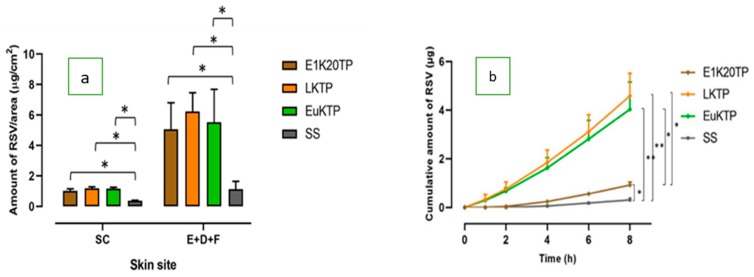
The skin penetration and permeation profiles of RSV from E1K20TP, LKTP, and EuKTP formulations: (**a**) the distribution of RSV in the SC and in the area of E+D+F; (**b**) the cumulative amount of RSV after 8 h of permeation through the skin (± SEM; 5–6 replications; *P < 0.05; **P < 0.01).

The nanoformulations all significantly increased the penetration of RSV in the SC and deeper skin tissues compared to the RSV-saturated solution, regardless of the terpene content (Figure 5a). They also significantly increased the RSV permeation through piglet skin to the receptor compartment (Figure 5b; Table 5). The nanoformulations containing limonene and eucalyptol (LKTP and EuKTP) provided a significantly greater RSV permeation than E1K20TP (*P* < 0.05; Figure 5b), with RSV enhancement ratios of 10–13-fold compared to 2.8-fold, respectively.

### 3.5. Stability of Nanoformulations

**Figure 6:**
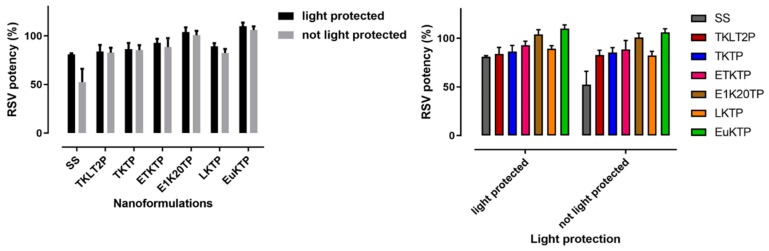
% RSV remaining after a 1-month storage at 22–25 °C (mean ± SD; *n* = 4).

The nanoformulations maintained at 22–25 °C for 1 month were stable, with the RSV % remaining in the range 82.69 ± 5.02% to 109.98 ± 3.72%, regardless of protection from light (Figure 6). The nanoformulations maintained their physical and chemical stability at 2–5 °C for up to approximately 6 months of storage, with the RSV % remaining over 89% (Table 6). The saturated solution of RSV in PBS pH 6 was relatively stable for one month at 22–25 °C when protected from light, but was reduced significantly without light protection (81.01 ± 1.05 % and 52.43 ± 13.55 %, respectively).

## 4. Discussion

Surfactant-containing nanoformulations were chosen as they offer the advantages of a simplicity of fabrication, excellent solubilizing capacity, and attractive appearance. The quality criteria set for the product were that these RSV nanoformulations must be clear (attractive appearance), stable (physically and chemically), safe, and effective (good RSV delivery into the skin). The clarity of the formulations can also be further used as a visual tool ensuring that the RSV is solubilised completely in one phase of the nanoformulation. Part of the strategy of this study was to develop nanoemulsions that required less oil, surfactant, and cosurfactant but that maintained an adequate RSV solubility with a high thermodynamic activity [27], good physical qualities, and enhanced skin penetration and permeation of RSV. Minimising solvent concentrations has a range of advantages, including reducing the potential for skin irritation, as well as minimizing the cost and impact on the environment.

Overall, in this study the nanoformulations containing RSV met the QTPP, with differences in the level of performance based on the choice and concentration of formulation excipients. The mechanisms underlying skin penetration/permeation enhancement of any applied drug include increasing the drug thermodynamic driving force, improving the drug solubility-partition in the SC, escalating the intercellular lipid fluidization, and preserving/increasing the SC hydration [28]. These mechanisms were considered in the design of the nanoformulations in this study.

Triacetin (glyceryl triacetate) was selected as the oil phase because it provides a good clarity, low viscosity, and good compatibility in the nanoemulsion system, providing a clear-single phase system. It is a commonly used solvent, solubilizer, and emulsion oil phase [29] that is categorised as GRASE (Generally Recognised as Safe and Effective) by the U.S. Food and Drug Administration (FDA) [30]. Triacetin has also shown a skin penetration enhancement effect [31]. The selected surfactant, Kolliphor^®^ RH 40 (polyoxyl 40 hydrogenated castor oil), is a non-ionic solubilizer and emulsifying agent [32,33,34]. Its hydrophobic moiety is a combination of glycerol polyethyleneglycol hydroxystearate and fatty acid glycerol polyglycol esters, while the hydrophilic moiety is a combination of polyethylene glycols and glycerol ethoxylate [33]. The HLB (hydrophilic-lipophilic balance) of this surfactant is between 14–16 [33] and thus appropriate for o/w nanoemulsions. Transcutol^®^ P, a high purity grade of diethylene glycol monoethyl ether (DEGEE), was selected as the cosurfactant because it is an excellent and safe hydroalcoholic solubilizer and skin permeation enhancer without compromising skin integrity [28,35,36,37,38]. PBS pH 6 was used as the aqueous phase to maintain the stability of RSV [12] and to support skin compatibility [25]. 

The capacity of nanoformulations to enhance the penetration and permeation of RSV in the skin was evaluated as the endpoint parameter of the skin-targeted formulation development. The penetration of RSV into the skin was assessed separately as RSV deposition in the SC, and in the combined areas of epidermis, dermis, and follicles (E+D+F). The permeation of RSV through the skin was also assessed by measuring the cumulative amount of RSV in the receptor phase, and calculating the steady state flux and lag-times. Due to the lack of availability of human skin, newborn pig skin was used as a previously validated human skin surrogate [39,40,41,42,43]. 

With the exception of TKLT2P, all nanoemulsion formulations provided a significantly better skin delivery of RSV than the saturated aqueous solution of RSV, with enhancement ratios (ER: based on the steady state flux) ranging from 2–12 (Table 5). The skin permeation results demonstrate that the nanoformulations with a lower solubility of RSV in the formulation have a higher RSV penetration and permeation into and through the skin. This follows Higuchi’s observations that a permeant with a lower solubility in the vehicle is more thermodynamically active and therefore more likely to partition from the vehicle to the skin [44,45]. Indeed, Higuchi [44] suggested that a more-than-needed capacity of a vehicle in solubilizing the drug can actually reduce the delivery rate of the drug from the vehicle to the skin. 

Our results also demonstrated that the inclusion of terpenes in the nanoemulsion formulation can increase the skin delivery of RSV (Table 5). Terpenes have been previously demonstrated to enhance penetration and permeation through SC intercellular lipid disruption by creating polar microchannels that facilitate an increase in permeant diffusivity [46,47,48,49]. Eugenol was selected based on its medium viscosity, which was expected to facilitate good product spreadability on the skin, and based on its pleasant aroma. The addition of eugenol (5%) significantly increased the amount of RSV in the (E-D-F) and permeation through the skin compared to the TKTP nanoemulsion of the same vehicle composition. This increase in skin delivery is likely due to the established mechanism of the interaction of eugenol with the SC lipids in increasing lipid fluidization [50]. The removal of triacetin from the nanoformulation resulted in a decrease in the RSV skin penetration and permeation, suggesting that eugenol and triacetin act synergistically to increase the RSV skin delivery. As triacetin is a known penetration enhancer with the same suggested SC lipid fluidization mechanism as terpenes [51,52], it is possible that both can work together to facilitate RSV diffusivity.

The effects of three terpenes-based nanoformulations (Eugenol - E1K20TP, D-limonene – LKTP, and Eucalyptol - EuKTP) were further investigated. The physical characteristics and RSV distribution in the skin were similar, but the permeation of RSV through the skin was higher for LKTP and EuKTP than for E1K20TP. This was not associated to differences in thermodynamic activity, as the solubility of RSV in these nanoformulations was similar regardless of the terpene that was present (Table 2). We suggest that the difference in the lipophilicity of terpenes contributed to their effectiveness in enhancing the RSV skin permeation. The ER for the RSV steady state flux was 12.6, 10, and 2.8 for the nanoemulsions containing eugenol, eucalyptol, and D-limonene, which correlated with the lipophilicity (log Po/w) of the three terpenes (3.4, 2.5, and 2, respectively [53,54,55]). This correlation was previously shown by El Kattan et al. [56,57] who reported a positive correlation between the lipophilicity of the terpenes and the cumulative amount of hydrocortisone permeating through hairless mouse skin. They showed that this was at least partly due to a better drug partitioning into the skin with a higher lipophilicity of the terpenes [56,57]. 

As RSV has a poor stability in the presence of light, one of the quality criteria set for the formulation development was an enhanced RSV stability during storage. This was achieved as all nanoformulations demonstrated a good stability at room temperature (22–25 °C) for one month and in the fridge for 6 months. Nanoformulations stored at 22–25 °C were stable regardless of the light exposure. This was in contrast to an RSV-saturated aqueous solution which showed a 20 to 50% reduction in the RSV content at one month when protected from and exposed to light, respectively. This demonstrates the capacity of the nanoformulations to protect RSV and is likely due to their ability to encapsulate the RSV within their globular structure [58]. 

In summary, we successfully developed self-assembling, stable, and physically suitable RSV nanoformulations to enhance the penetration and permeation of RSV into and through the skin, which met the QTPP. These nanoformulations could be used to facilitate the skin delivery of RSV and to potentially exert an antioxidant effect in the skin. The effective transdermal delivery of RSV has potential benefits as the antioxidant effects could reduce skin damage following exposure to UV light and slow the signs of skin aging.

## Figures and Tables

**Table 1:** Nanoformulations (all as % *w*/*w*).

Composition	Formula						
	TKLT2P	TKTP	ETKTP	E5K30TP	E1K20TP	LKTP	EuKTP
Triacetin	25.7	5	5	-	-	-	-
Kolliphor^®^ RH 40	25.7	20	30	30	20	20	20
Labrasol^®^	12.8	-	-	-	-	-	-
Transcutol^®^	12.8	10	10	10	10	10	10
Eugenol	-	-	5	5	1	-	-
D-limonene	-	-	-	-	-	1	-
Eucalyptol	-	-	-	-	-	-	1
PBS pH 6	23	65	50	55	69	69	69

**Table 2:** Physical characteristics of the RSV nanoformulations (*n* = 3).

Formula	Appearance			RSV Solubility (mg/mL)	Viscosity (dPas) *	Refractive Index *
	Clarity	Single Phase	Colour			
TKLT2P	translucent	✓	Light brown	177.16 ± 25.95	0.790 ± 0.070	1.4253 ± 0.0007
TKTP	transparent	✓	Light brown	44.77 ± 4.16	0.107 ± 0.021	1.3769 ± 0.0005
ETKTP	transparent	✓	Light brown	n.a	1.627 ± 0.136	1.4021 ± 0.0002
E5K30TP	transparent	✓	Light brown	n.a	1.280 ± 0.053	1.3850 ± 0.0033
E1K20TP	transparent	✓	Light brown	34.09 ± 1.13	0.097 ± 0.006	1.3747 ± 0.0003
LKTP	transparent	✓	Light brown	35.46 ± 1.60	0.083 ± 0.015	1.3732 ± 0.0011
EuKTP	transparent	✓	Light brown	37.25 ± 3.68	0.093 ± 0.015	1.3918 ± 0.0329

Note: * measurements conducted on blank nanoformulations. pH = 6 for all formulations due to the buffer.

**Table 3:** The globule size and PDI measurement of the blank nanoformulations (*n* = 3).

Formula	Globule Size (nm)	PDI
TKLT2P	14.30 ± 0.05	0.229 ± 0.010
TKTP	13.72 ± 0.40	0.106 ± 0.072
ETKTP	13.97 ± 0.18	0.055 ± 0.007
E5K30TP	13.60 ± 0.07	0.046 ± 0.008
E1K20TP	13.84 ± 0.01	0.071 ± 0.010
LKTP	15.73 ± 0.07	0.117 ± 0.003
EuKTP	14.54 ± 0.04	0.091 ± 0.044

**Table 4:** The skin distribution of RSV from nanoformulations (mean ± SEM; *n* = 5–6). ER_SD_ calculated as the ratio of the total mean amount of RSV in SC and E+D+F from nanoformulations to the saturated aqueous solution.

Formula	RSV Distribution in the Skin		ER_SD_
	SC	E+D+F	
TKLT2P	0.805 ± 0.208	2.915 ± 1.523	2.48
TKTP	1.998 ± 0.383	5.359 ± 0.845	4.90
ETKTP	2.342 ± 0.269	12.000 ± 3.598	9.55
E5K30TP	2.104 ± 0.297	5.914 ± 1.169	5.34
E1K20TP	1.022 ± 0.129	5.059 ± 1.744	4.05
LKTP	1.190 ± 0.092	6.234 ± 1.231	4.94
EuKTP	1.172 ± 0.085	5.526 ± 2.160	4.46
SS	0.378 ± 0.025	1.124 ± 0.519	1.00

**Table 5:** The experimental data for the RSV skin permeation parameters in nanoformulations (mean ± SEM; n = 5–6). ER_FLX_ was calculated based on the ratio of the steady state flux of the nanoformulation to the steady state flux of the saturated aqueous solution.

Formula	Cumulative Amount (µg)	Flux (µg/cm^2^/h)		Lag Time (h)	ER_FLX_
		Steady State Flux (*J_ss_*)	Maximum Flux (*J_max_*)		
TKLT2P	0.278 ± 0.086	0.038 ± 0.010	0.339 ± 0.091	2.330 ± 0.248	0.75
TKTP	0.853 ± 0.091	0.103 ± 0.006	0.227 ± 0.013	1.711 ± 0.605	2.01
ETKTP	2.973 ±1.051	0.358 ± 0.125	n.a	1.195 ± 0.280	6.98
E5K30TP	2.017 ± 0.954	0.116 ± 0.059	n.a	0.636 ± 0.188	2.27
E1K20TP	0.918 ± 0.126	0.142 ± 0.017	0.258 ± 0.029	2.689 ± 0.224	2.76
LKTP	4.585 ± 0.936	0.647 ± 0.103	1.191 ± 0.209	1.252 ± 0.715	12.61
EuKTP	4.036 ± 1.125	0.510 ± 0.153	0.920 ± 0.277	1.143 ± 0.164	9.95
SS	0.309 ± 0.074	0.051 ± 0.009	0.051 ± 0.009	3.185 ± 0.176	1.00

**Table 6:** The RSV stability during the long-term storage (5–8 months) at 2–5 °C and protected from light.

Formula	Duration of Storage (month)	Physical Stability			Chemical Stability (%)
		Clarity	Single Phase	Tendency of Darker Appearance	
TKTP	8	transparent	✓	+	98.73 ± 4.00
ETKTP	5	transparent	✓	++	89.25 ± 1.70
E1K20TP	6	transparent	✓	++	93.39 ± 8.17
LKTP	6	transparent	✓	+	107.02 ± 8.73
EuKTP	6	transparent	✓	+	108.41 ± 4.62
